# LIFESTYLE BEHAVIORS AND RACIAL DIFFERENCES IN ALZHEIMER’S DISEASE RISK

**DOI:** 10.1093/geroni/igz038.1748

**Published:** 2019-11-08

**Authors:** Roger Wong

**Affiliations:** Washington University in St. Louis, Saint Louis, Missouri, United States

## Abstract

There is substantial evidence indicating that among all racial groups, Blacks have the highest Alzheimer’s disease (AD) risk. Despite the wide support for this disparity, the reasons for these racial differences in AD risk remain unclear. The purpose of this study was to examine how lifestyle behaviors (physical activity, smoking, and social contacts) mediate and moderate the relationship between race and AD risk among Black and White older adults. This study used seven annual waves (2011-2017) of prospective data from the National Health and Aging Trends Study (NHATS), a large nationally representative U.S. sample of older adults. At each wave, physical activity was measured as whether they engaged in vigorous physical activities; smoking was measured as whether they were cigarette smokers; and social contacts was measured as whether they visited friends/family outside of their home. The dependent variable was age of AD diagnosis. Multivariate analyses were conducted using the Cox proportional hazards model. Blacks had a 1.3 times significantly higher risk for AD compared to Whites (Hazard Ratio [HR]=1.31, p=.03). After controlling for lifestyle behaviors as a mediator, racial differences in AD risk were attenuated and no longer significantly different between Blacks and Whites (HR=1.05, p=.74). For the moderation model, interactions between race and each lifestyle behavior generated no statistically significant results. Our findings indicate lifestyle behaviors contribute to racial differences in AD risk between Blacks and Whites. Future research is needed among Black populations to identify specific lifestyle behaviors that are especially protective against AD as targets of intervention.

